# Microsatellite alteration and immunohistochemical expression profile of chromosome 9p21 in patients with sporadic renal cell carcinoma following surgical resection

**DOI:** 10.1186/s12885-016-2514-8

**Published:** 2016-07-27

**Authors:** Ismail El-Mokadem, Alison Lim, Thomas Kidd, Katherine Garret, Norman Pratt, David Batty, Stewart Fleming, Ghulam Nabi

**Affiliations:** 1 Academic Section of Urology, Division of Cancer Research, Ninewells Hospital, University of Dundee, Dundee, DD1 9SY UK; 2 Department of Cytogenetic, Ninewells Hospital, University of Dundee, Dundee, DD1 9SY UK; 3 Department of Pathology, Ninewells Hospital, University of Dundee, Dundee, DD1 9SY UK

**Keywords:** Kidney cancer, Genetics, Microsatellite analysis, Chromosome 9p

## Abstract

**Background:**

Long-term prognostic significance of loss of heterozygosity on chromosome 9p21 for localized renal cell carcinoma following surgery remains unreported. The study assessed the frequency of deletions of different loci of chromosome 9p along with immunohistochemical profile of proteins in surgically resected renal cancer tissue and correlated this with long-term outcomes.

**Methods:**

DNA was extracted from renal tumours and corresponding normal kidney tissues in prospectively collected samples of 108 patients who underwent surgical resection for clinically localized disease between January 2001 and December 2005, providing a minimum of 9 years follow-up for each participant. After checking quality of DNA, amplified by PCR, loss of heterozygosity (LOH) on chromosome 9p was assessed using 6 microsatellite markers in 77 clear cell carcinoma. Only 5 of the markers showed LOH (D9S1814, D9S916, D9S974, D9S942, and D9S171). Protein expression of p15(INK4b), p16(INK4a), p14(ARF), CAIX, and adipose related protein (ADFP) were demonstrated by immunostaining in normal and cancer tissues. Loss of heterozygosity for microsatellite analysis was correlated with tumour characteristics, recurrence free, cancer specific, and overall survival, including significance of immunohistochemical profile of protein expressions.

**Results:**

The main deletion was found at loci telomeric to CDKN2A region at D9S916. There was a significant correlation between frequency of LOH stage (*p* = 0.005) and metastases (*p* = 0.006) suggesting a higher LOH for advanced and aggressive renal cell carcinoma. Most commonly observed LOH in the 3 markers: D9S916, D9S974, and D9S942 were associated with poor survival, and were statistically significant on multivariate analysis. Immunohistochemical expression of p14, p15, and p16 proteins were either low or absent in cancer tissue compared to normal.

**Conclusions:**

Loss of heterozygosity of p921 chromosome is associated with aggressive tumours, and predicts cancer specific or recurrence free survival on long-term follow-up.

**Electronic supplementary material:**

The online version of this article (doi:10.1186/s12885-016-2514-8) contains supplementary material, which is available to authorized users.

## Background

Microsatellite alterations in tumor DNA relative to normal are potential genetic prognostic markers for RCC. The most common method that is used to detect these alterations relies on assays which examine two distinct alleles at a particular location, a condition known as heterozygosity. A normal tissue specimen is compared with a tumour sample from the same patient, and DNA loss is identified if one of the alleles present in the normal specimen is lost in the tumour, a condition termed as loss of heterozygosity (LOH). Loss of heterozygosity (LOH) at chromosome 3p and inactivation of the von Hippel-Lindau tumor suppressor (VHL) genes are known to have association with ccRCC [1]. Several studies, including those of our own group have shown that chromosome 9p has a critical region of loss in renal cell carcinoma [2–5]. Significance of these molecular genetic events on 9p, in particular, their correlation with the clinical outcomes following treatment are not fully understood. Investigators previously used microsatellite markers to identify loss of various loci on 9p chromosome in renal cell cancer with various frequencies of microdeletions. Grady et al. [6] reported 73, 40, and 23 % loss of heterozygosity at a minimum of 1, 3 and at 4 or more loci respectively in a small series of 60 cases. This study, however, did not report clinical outcomes or immunohistochemical expression of the cohort, hence, clinical implications of their findings remain unknown.

Fluorescent in situ hybridization studies have shown loss of chromosome 9p in renal cancer carries a poor prognosis [5], but mapping studies on 9p are only a few and none with long-term outcomes. Frequent allelic loss has been reported in lung, ovarian, bladder, oral, and head-and-neck cancers on chromosome 9p21 [2, 7, 8]. The locus on 9p encodes for three cell-cycle inhibitory proteins: p14^ARF^, which is encoded by an alternative reading frame of *CDKN2A*, p15^INK4b^, which is encoded by *CDKN2B*, and p16^INK4a^, which is encoded by *CDKN2A*. The p14^ARF^ protein activates the key checkpoint protein p53, thereby inducing either cell-cycle arrest (both in G_1_ and G_2_) or apoptosis. Both p15^INK4b^ and p16^INK4a^ are able to induce cell-cycle arrest in G_1_ by inhibiting the cyclin-dependent kinases CDK4 and CDK6 to inactivate the retinoblastoma (*RB1*) family of tumor suppressor proteins [9, 10].

In the present study, we report microsatellite alterations (LOH) on chromosomes 9p using different markers along with comprehensive immunohistochemical analysis and correlate this with the long-term outcomes of patients with ccRCC after surgical excision with curative intent.

## Methods

### Study cohort

The Tayside Urological Cancer Network (TUCAN) databases were searched for consecutive patients who underwent radical nephrectomy for clinically localized Renal Cell Carcinoma (RCC) between Jan. 2001 and Dec. 2005. This is similar cohort described previously [5]. Patients with cytoreductive nephrectomy were excluded from this study. The period was chosen so that a minimum of 9 years follow-up for each patient is obtainable. Ethics approval (Ref. 12/ES/0083) was obtained from the Tayside Research and Ethics committee. A single pathologist (SF) with special interest in renal cancer reviewed all the samples using pathology number identifier for each patient. Patients were followed up using standard department similar to already described in our previous publications [11, 12].

### DNA extraction from paraffın-embedded tissues

DNA was isolated from the paraffin-embedded tissues according to the Qiagen EZ1 BioRobot method [13]. This extraction protocol relies on the use of magnetic particles which bind to the DNA and are subsequently removed from the surrounding tissue by a separate magnetic source. Briefly the curls of tissues were extracted with 1 ml of xylene to remove the paraffin and then treated twice with 1 ml of 95 % ethanol. After air-drying, tissue pellets were digested in 180 μl of tissue lysis buffer containing 0.1 M Tris–HCl (pH 8.0), 1 mM EDTA, 0.5 % Tween 20 and 20 μl proteinase K at 56 °C overnight. Digested mixtures were heated at 90 °C for 5 min to inactivate proteinase K. Supernatants were aliquoted to fresh Eppendorf tubes, then diluted to a final volume of approximately 400 μl by addition of 200 μl of 10 mM Tris–HCl (pH 8), 1 mM EDTA buffer and stored at −20 °C until use.

### PCR-LOH analysis

DNA was amplified using PCR and analysed for LOH on chromosome 9 using 6 different polymorphic DNA markers. Briefly 50 to 100 ng of DNA (1 μl) were amplified by PCR in 25 μl of solution containing 2.5 mM each of dATP, dGTP, dCTP, and dTTP; 1 μCi [1-^32^P] dCTP; 5 μM forward and reverse primers; 0.1 μl (0.5U) HotStar Taq Start antibody (Clontech, Palo Alto, CA); and 1 unit of Taq polymerase (Perkin-Elmer, Foster City, CA) in 10 PCR buffer [10 mM Tris–HCl, 50 mM KCl and 1.5 mM MgCl_2_ at pH 8.3 (Perkin-Elmer)]. PCR was started at 95 °C for 15 min, followed by 35 cycles of denaturing (95 °C for 40 s), annealing (55° for 40 s), and chain extension (72 °C for 40 s), followed by 7 min at 8 °C and 1 min at 4 °C. The primer sequence and annealing temperature for each pair of primers are listed in Table 1. The PCR product (0.5 μl) was denatured in deionised fomamide and the fluorescent markers were analysed by capillary electrophoresis. Determination of loss of heterozygosity (LOH) was carried out using GeneMarker® software V2.4.0 (SoftGenetic®, State College, PA 16803, USA). For informative cases, allelic loss (or possible allelic imbalance) was scored if one allele was significantly decreased in tumor DNA (40 %) compared with the same allele in normal control DNA.

**Table 1:** Shows the sequences of microsatellite markers used in the study

Primers	Sequence
D9S916	Forward 5’- gatgtccagttgtcccttcataa -3’
	Reverse 5’- atagactgccaaatttttggacc -3’
D9S974	Forward 5’- cctggtctggatcataaaatgaa -3’
	Reverse 5’- tgtggaaattttctgtctggttc -3’
D9S942	Forward 5’- aagcaagattccaaacagtaaaca -3’
	Reverse 5’- ttcgtttcacttttgagttttcc -3’
D9S1814	Forward 5’- tgtcagtggtatttacctttttgg -3’
	Reverse 5’- cagaaggtcagtaggttcacagg -3’
D9S171	Forward 5’- agctaagtgaacctcatctctg -3’
	Reverse 5’- tgattgttaataaagtagcccc -3’

### Immunohistochemistry

Immunostaining was used to identify p14, p15, p16, CAIX, and ADFP protein expressions in renal cancer and normal samples. Antigen retrieval and de-paraffinisation was performed using DAKO EnVision™ FLEX Target Retrieval solution (high pH) buffer in a DAKO PT Link. Immunostaining using DAKO EnVision™ FLEX system on a DAKO Autostainer Link48 was carried out according to manufacturer’s protocol. Sections were incubated with primary antibody for 30 mins. DAKO substrate working solution was used as a chromogenic agent for 2 × 5mins and sections were counterstained in EnVision™ FLEX hematoxylin. Sections known to stain positively were included in each batch and negative controls were prepared by replacing the primary antibody with DAKO antibody diluent. The primary investigator, blinded to clinical outcome, FISH, and microsatellites results, scored TMA slides for p14, p15, and p16 antibodies using a Nikon Eclipse E600 microscope. The scoring consisted of 2 different techniques based on the intensity of cytoplasmic immune-staining on a 4-point scale (Table 2) and the percentage of nuclei with immune-staining in each core based on 3-point scale (Table 2). Both systems were applied for *p14, p15, and p16* antibodies. In contrast, only the 4-point scale for cytoplasmic immune-staining was employed for scoring ADFP and CAIX antibodies. The scoring process was supervised and confirmed by a specialist consultant renal pathologist.

**Table 2:** Shows methodology of scoring of immunohistochemistry for various proteins expressed in normal and renal cancer

Cytoplasm staining	Score	Interpretation
	X	Core loss
	N	Normal renal tissue
	0	No uptake of stain
	1	Diffusely or focally weak uptake of the stain
	2	Diffusely or focally moderate uptake or focally intense
	3	Diffusely intense
Nuclear staining	Score	Percentage Nuclei Stained %
	1	0–10 %
	2	>10 but <50 %
	3	≥50 %

Non-integral values were rounded up to close integral figure

### Statistical analysis

The number of cores and nuclei scored by each observer was compared. The level of interobserver agreement upon validity of scoring and deletion status per represented block of tumour was assessed using Kappa statistical analysis. The clinicopathological data was compared based on LOH status. Proportions between categorical variables were compared using Fisher exact and Pearson chi-square tests, as appropriate. The survival time was summarised using median and interquartile ranges (IQR). Other continuous variables were summarised as mean and standard deviation (SD), and compared using Student-*t* tests or Mann–Whitney *U* test as appropriate.

The Kaplan-Meier method was used to estimate RFS and DSS based on LOH status and other variables. The log-rank test was used to compare the survival differences between the groups.

A Cox-proportional hazard model was used to assess the correlation between prognostic variables and recurrence, and RCC-specific mortality. Multivariate analysis was performed for DSS and RFS after excluding the insignificant variables on univariate analysis. For DSS, 2 models were generated. A backward selection manner with the likelihood ratio criterion (for entry and removal: *p* ≤ 0.05 and *p* > 0.1 respectively) and rank of elimination was used to identify the most significant variables to be entered in a final model for RFS and 2 final prognostic models for DSS. Statistical analysis was performed using IBM® SPSS® – version 21, with all tests being two-tailed and *p* < 0.05 considered statistically significant.

## Results

**Fig. 1:**
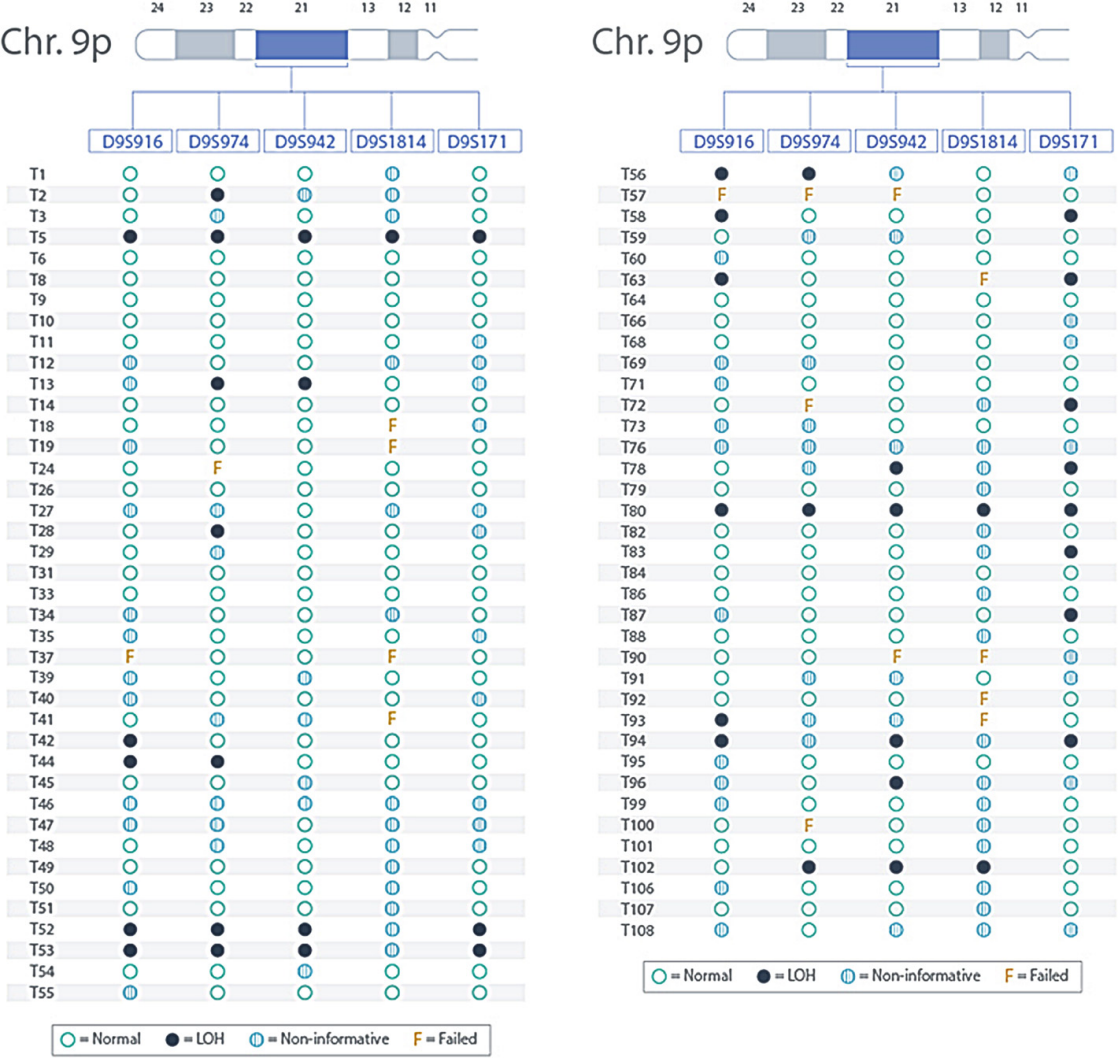
**a** and **b** Show outcome of microsatellite status at chromosome 9p using 5 different markers. Clear circle represents normal, black circle represents loss of heterozygosity (LOH), crossed circle represents non-informative and F represents failed

**Fig. 2:**
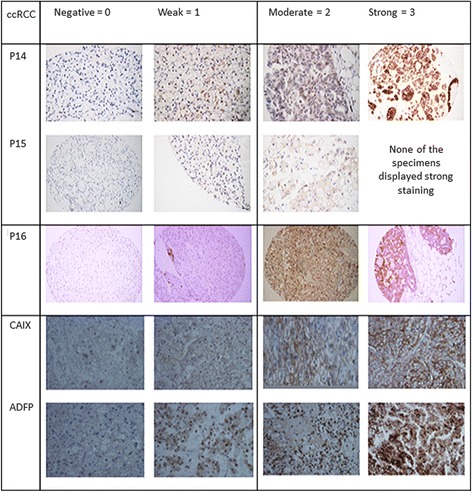
Shows an example of variation in immunohistochemical staining pattern (negative to strong) seen in normal and cancer tissues for various proteins with common origin from chromosome 9p. Overall, normal tissue strong staining pattern compared to corresponding cancerous tissue. Methodology described in Table 2 was used to quantify the staining pattern of immunohistochemical expression

There were 77 ccRCC tumour/normal interpretable pairs for LOH analysis. Figure 1a and b show different loci on chromosome 9 of renal cancer tissues and summarizes microsatellite analysis profile. LOH was identified with at least for 1 marker in 20 of 77 (26 %) cases. Most allelic deletions were detected, telomeric to CDKN2A region at D9S916, with 11 out of 52 informative tumours (21 %) displaying LOH. For the 2 microsatellites within CDKN2A coding region, ten out of 58 informative cases (17 %) and 9 out of 62 informative cases (14.5 %) displayed LOH at D9S974 and D9S942 respectively. In contrast, centromeric to CDKN2A coding region, D9S1814 showed LOH in 3 out of 38 cases (7.8 %), which was the lowest rate of LOH as well as informative cases out of the 5 primers. D9S171 (9p13) showed allelic deletion in 11 out of 57 informative tumours (19 %). A summary of the renal cell carcinoma cases with grade, stage, and LOH on chromosome 9 is given in Table 3. Additional file 1: Figure S1 shows the representative LOH at chromosome 9p in human renal cancer. There were deletions on loci D9S162, D9S1748, D9S171, D9S270, and D9S153 of chromosome 9p. Figure 2 is an example showing pattern of protein expression on immunohistochemistry profile seen. The level of expression (none to strong) of different proteins was compared between ccRCC and control normal renal tissues. The levels of expression of p14, p15, and p16 in ccRCC were statistically significantly lower compared to their levels of expression in normal renal tissue (Table 4). On the other hand, there was no difference in the degree of expression between ADFP and CAIX expression between ccRCC and normal renal tissue.

**Table 3:** Correlation between loss of heterozygosity of chromosome 9p and various tumour characteristics in clinically localized renal cell carcinoma

		D9S916			D9S974			D9S942			D9S1814			D9S171		
Variables		Normal	LOH	*p*=	Normal	LOH	*p*=	Normal	LOH	*p*=	Normal	LOH	*p*=	Normal	LOH	*p*=
Tumour size	Mean	5.3	4.6	0.93	4.8	6.4	0.76	4.8	6.4	0.17	4.8	6.4	0.77	5.1	5.3	0.66
	SD	(±3.2)	(±2.1)		(±2.9)	(±3.4)		(±2.9)	(±3.4)		(±2.9)	(±3.4)		(±3.2)	(±2)	
T stage	pT1	20	4	*0.005*	28	2	*0.004*	29	2	*0.003*	19	0	*0.028*	22	5	0.42
	pT2	12	0		7	1		11	0		8	0		12	0	
	pT3	9	5		13	5		13	6		7	3		12	5	
	pT4	0	2		0	2		0	1		1	0		0	1	
	pT1/2	32	4	*0.02*	35	3	*0.02*	40	2	*0.004*	27	0	0.02	34	5	0.084
	pT3/4	9	7		13	7		13	7		8	3		12	6	
Metastasis	N0M0	38	8	0.1	46	6	*0.006*	50	5	*0.006*	33	2	0.224	42	8	0.12
	N + M+	3	3		2	4		3	4		2	1		4	3	
Fuhrman Grade	G1/G2	16	4	1	18	2	0.47	20	2	0.47	14	1	1	18	3	0.73
	G3/G4	25	7		30	8		33	7		21	2		28	8	
Tumour necrosis	Present	10	3	1	14	3	1	16	1	0.42	10	0	0.55	14	3	1
	Absent	29	7		33	6		35	7		23	3		30	7	
Sarcomatoid change	Present	1	3	*0.02*	2	2	0.12	4	1	0.53	4	1	0.37	3	2	0.23
	Absent	38	7		45	7		47	7		29	2		41	8	
Microvascular invasion	Present	10	2	1	11	3	0.68	13	3	0.67	9	1	1	12	3	1
	Absent	28	8		36	6		38	5		23	2		31	7	
Renal Vein invasion	Present	6	2	1	8	2	0.67	8	2	0.62	3	1	0.32	7	2	1
	Absent	31	8		37	7		41	6		28	2		34	8	
Pelvicalyceal invasion	Present	4	0	0.56	6	1	1	6	1	1	2	1	0.25	7	0	0.32
	Absent	33	10		39	8		43	7		29	2		34	10	
Renal sinus invasion	Present	3	4	*0.03*	5	4	*0.03*	5	4	*0.016*	3	2	0.068	5	4	0.06
	Absent	34	6		40	5		44	4		23	1		36	6	
*SSIGN* score	0–2	17	5	0.16	21	3	0.26	22	2	0.29	16	1	0.011	17	4	0.43
*S – Stage*	03–Apr	4	0		7	0		6	1		4	0		4	0	
*SI – Size*	05–Jun	11	1		9	2		11	2		3	1		10	3	
*G – Grade*	07–Sep	4	2		6	2		8	1		8	0		8	1	
*N - Necrosis*	≥10	1	2		2	2		2	2		0	1		2	2	

Numbers in columns under normal and LOH represent number of patients

**Table 4:** Percentage and expression level of nuclear immunostaining for various proteins in ccRCC vs normal renal tissue

Proteins	Percentage of immunostaining	Clear cell RCC	Normal renal tissue	Pearson Chi-square (*p*=)
P14	<10 %	70	1	<0.001
	11–50 %	8	6	
	>50 %	4	13	
Mean expression (SD)		1.336 (0.56)	2.714 (0.42)	
P15	<10 %	76	20	<0.03
	11–50 %	4	0	
	>50 %	0	0	
Mean expression (SD)		0.970 (0.58)	1.631 (0.36)	
P16	<10 %	72	6	<0.001
	11–50 %	9	10	
	>50 %	1	4	
Mean expression (SD)		1.441 (0.62)	1.809 (0.31)	

### Clinicopathological significance of LOH and proteins expression

**Fig. 3:**
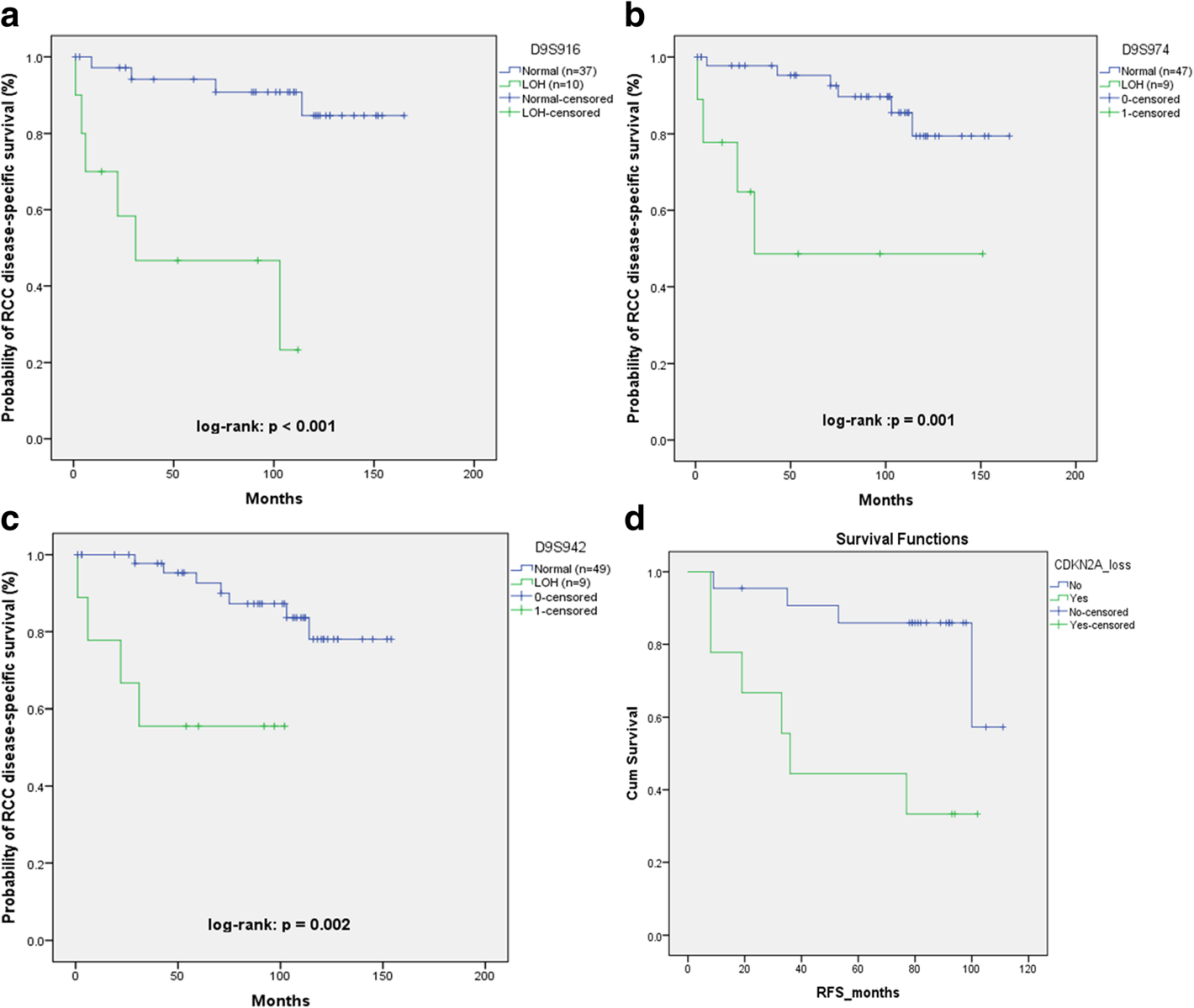
Number of figures in this group show disease specific survival and recurrence free survival in patients with loss of heterozygosity for various markers. **a** Shows poor survival in patients with loss of heterozygosity for D9S916 **b** Shows poor survival in patients with loss of heterozygosity for D9S974; **c** Shows poor survival for patients with loss of heterozygosity for more than 1 loci on 9p21 region. **d** shows poor recurrence free survival of renal cancer patients following surgery with loss of heterozygosity on CDKN2A region

Cases displaying LOH at least in one marker on 9p21 were highly significantly associated with higher risk of RCC-specific death compared to cases with no allelic deletion (Fig. 3a-c). This was mostly observed with LOH in the 3 markers within this region with the highest number of informative cases: D9S916, D9S974, and D9S942. These were statistically significant on multivariate analysis (Table 5).

**Table 5:**
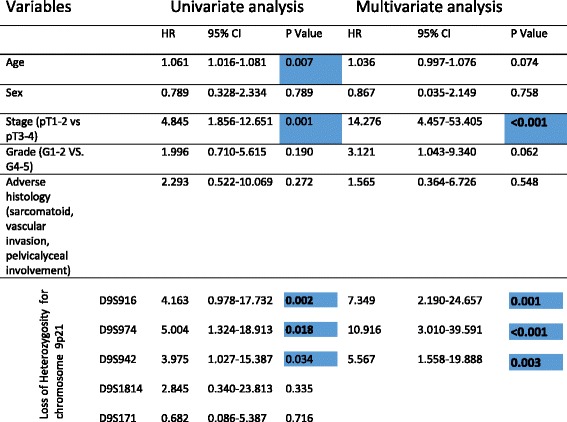
Shows summary of univariate and multivariate analysis. Note highlighted significant p-Values

Also, LOH at D9S171 (9p13) showed a trend towards worse prognosis, but did not reach statistical significance. The markers showed significant association with RCC recurrence (Log-rank: *p* = 0.014; Fig. 3d).

Out of the 65 informative cases, 59 had valid immunohistochemistry scoring. The level of expression of all protein markers correlated with the LOH at least in one of the microsatellites at chromosome 9p. The level of p16 cytoplasmic expression significantly correlated with LOH involving at least one of the microsatellites at 9p21 and was less in LOH cases compared with normal 9p status (Mann–Whitney U: *p* = 0.011).

**Fig. 4:**
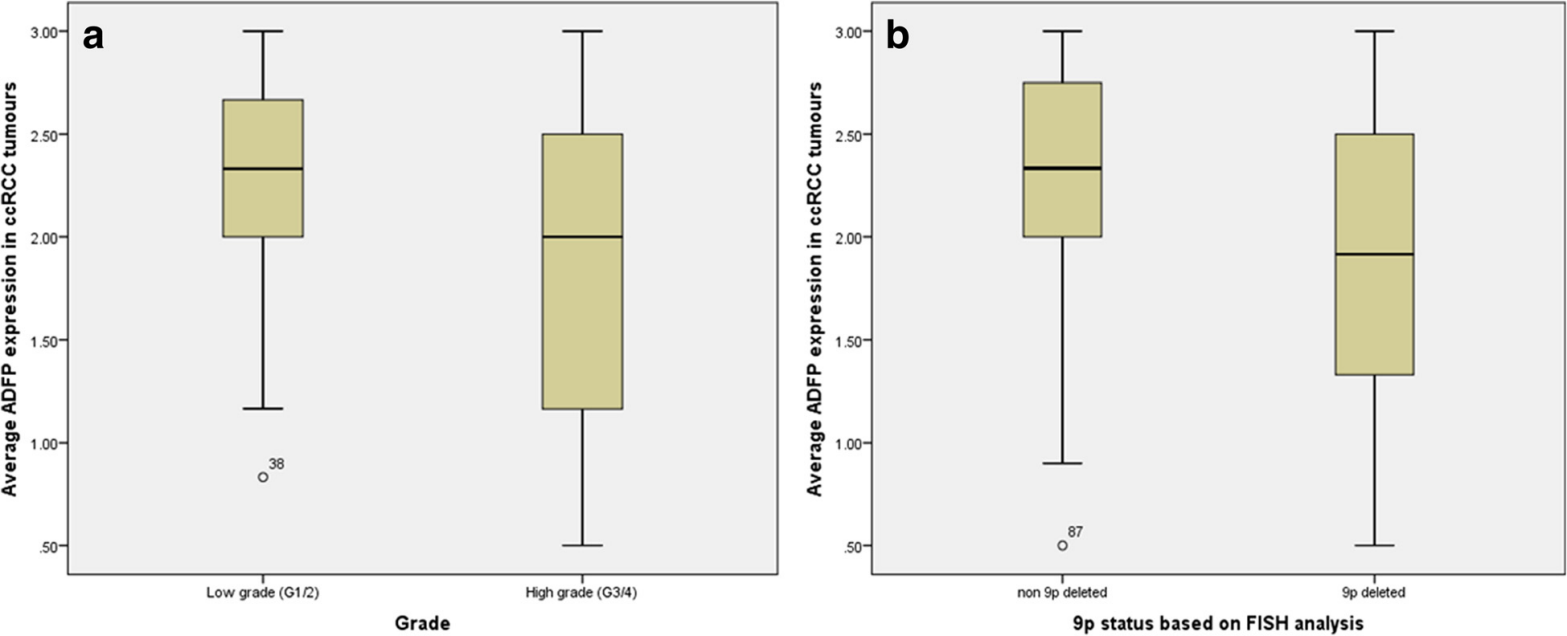
**a** Correlation between ADFP expression and histopathological grade of cancer (*p* = 0.021). **b** Also, see a good correlation between ADFP expression status and 9p deletion (*p* = 0.015)

**Fig. 5:**
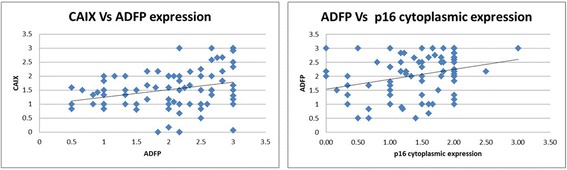
Correlation between ADFP expression and other two proteins (CAIX and P16) known to be in the region of CKDNA2 on chromosome 9p

A significant trend was seen between pathological stage and p14 cytoplasmic expression (*p* = 0.062; *r* = −0.169), p14 nuclear immunohistochemical staining (*p* = 0.07; *r* = −0.187). Similarly, positive correlation was observed with p15 nuclear expression (*p* = 0.09; *r* = 0.178). Lower ADFP expression was associated with higher Furhman grade (*p* = 0.021; *r* = −0.218; Fig. 4) tumours and the mean ADFP expression in 9p-deleted tumours was significantly lower compared to tumours with normal 9p status (1.87 and 2.25 respectively; *p* = 0.015). It was noted that the high levels of ADFP expression were associated with high levels of CAIX (*p* = 0.008; *r* = 0.293; Fig. 5) and p16 cytopalsmic expression (*p* = 0.004; *r* = 0.318).

## Discussion

This study investigated the association between loss of heterozygosity (LOH) in the region of chromosome 9p21, immunohistochemical expression of a number of proteins associated with the region, staging (lower vs. higher) of renal cancers and long-term outcome following resection of clinically localized renal cancer. Microsatellite analysis using five biomarkers investigated this relationship. The main allelic deletion was detected at loci telomeric to CDKN2A region at D9S916, with 11 out of 52 informative cases (21 %) displaying LOH. The frequency of LOH was directly linked to the stage (*p* = 0.005) and metastases (*p* = 0.006) of the tumours, indicating a higher frequency of LOH is associated with a more aggressive renal cell cancer. There was a significantly higher risk of cancer related death in patients showing LOH in at least one marker on 9p21 in comparison to those patients with no allelic deletion. Loss of heterozygosity at chromosome 9p was associated with p14^ARF^, *p15*^*INK4b*^, *p16*^*INK4a*^, adipose related differentiation protein (ADFP) and carbonic anhydrase IX expression. Loss of expression of p14^ARF^, p15^INK4b^, p16^INK4a^, and ADFP was significantly associated with aggressive tumours and poor prognosis. In addition to these key findings, the observations from this study contribute not only to estimation of the molecular pathophysiology renal cancer, but also to the improvement of clinical management of patients with renal cancer through the provision of better prognostic prediction.

LOH is an allelic imbalance that is used to detect tumour suppressor genes and genomic regions which may correlate with tumour grade, stage, and progression. Microsatellites have proved to be important genetic markers in analysing LOH, however, studying LOH using these markers has been limited due to the tedious nature of the genotyping procedure. There have been a number of studies investigating more effective ways of analysing LOH. Studies have shown that LOH and copy number aberrations (CNA) analysis by high-density single – nucleotide polymorphism (SNPs) arrays can be used effectively in identifying possible changes in genes [14]. In one study, polymerase chain reactions (PCR) were performed on a large number of SNPs for LOH and the amplified products of PCR were hybridised to a high-density oligonucleotide array. It was found that the relationship of LOH in cases directly corresponds with findings in genomic hybridization [15]. In a similar study, with larger numbers of SNP markers were shown to rapidly detect allelic imbalances; the results were validated by comparing them with analysis of the same tumours using the microsatellite allelotype method. The comparison successfully demonstrated consistent results between the two methods in identifying genomic imbalances [16].

Affymetrix 10 K SNP genome-wide mapping arrays have been studied and the results remain consistent with the existing literature, proving the effectiveness of SNP arrays analysis in detecting LOH and other chromosomal alterations. Toma et al. [17] confirmed that SNP array analysis is an important method of detecting novel microdeletions and may be involved in the progression of cc-RCC, as well as confirming larger chromosomal imbalances. The clinical application of LOH analysis have been investigated including its value in predicting prognosis and patient outcomes. Allelic loss using polymorphic microsatellites have been used to determine the progression of papillary renal cell carcinoma [18]. It is suggestive that LOH at chromosomes 8p12-21.1, 9p21, and 14q24.2-qter regions directly corresponds to higher tumour grade and pathological progression.

The importance of 9p status in cancer progression and patient outcomes has been of a matter great research interest for many years. However, the application of this genetic information has yet to progress to the clinical setting, and a few would question application of this information in healthcare. A recent systematic review [19] carried out to assess the quality of studies reporting the significance of 9p chromosomal abnormalities in RCC and to assess whether it could be used as a tool to predict oncological outcomes in RCC. Looking at 11 studies, including a total of 1431 patients, it was found that more work including standardization of techniques were needed to validate the clinical role of 9p status. Throughout the review, the studies lacked consistent interpretation of results and investigation into clinical application. The review called for more research into the 9p status predicting clinical outcomes, with a greater focus on the clinical application and a higher quality of research methodology and reporting.

Cancer staging and grading have long been the accepted methods of evaluating patient outcomes. Prognosis of renal cell cancer is conventionally predicted using staging and grading, relying on variables such as tumour morphology, tumour size, lymph node or distant metastatic spread and vascular invasion. However, there are limitations in using these traditional classifications as they are ineffective in accurately assessing which post-surgical patients are high risk of recurrence and most certainly fail to identify patients who would benefit from more closer follow up and adjuvant therapies. Using histopathological variables, the patient sub groups are too broad and there is variation within the individual sub groups which make predictive values of these measure inaccurate. It has been investigated by El-Mokadem et al. [5] as to whether interphase fluorescent in situ hybridisation (I-FISH) scoring can be used as an alternative for predicting disease-free survival and recurrence rates. Tissue microarrays were constructed from paraffin-embedded renal cell tumour tissue and I-FISH was used to determine 9p status. Patients were followed up for a median of 95 months and it was found that 9p deletion was an indicator of higher risk recurrence (*P* = 0.008) and RCC-related death (*P* = 0.001). It was also found that using 9p status with the SSIGN (stage, size, grade, and necrosis) score significantly increased the predictive accuracy from 87.7 to 93.1 %.

It has been found that 9p chromosomal deletions are linked with more aggressive cancers that have a higher risk of metastatic spread following nephrectomy for localized RCC [20]. Deletion in 9p can be commonly seen in a number of epithelial tumours. It is often present in larger tumours with higher grade and stage, however, it can also be found in less aggressive, smaller tumours, and 9p status still successfully predicts disease recurrence in these cases. To investigate LOH on 9p, 5 microsatellite markers of this region were identified and patients were followed up, looking at the association of 9p chromosome deletion with prognosis of patients after potentially curative nephrectomy. In a multivariate analysis, it was found that LOH at 9p21 chromosome achieved a statistical significance and accurately predicted survival or risk of RCC recurrence in patients following surgery.

Cyclin dependent kinases locus on chromosome 9p21 encodes for three cell cycle inhibitory proteins: p15INK4b encoded by CDKN2b, p16INK4a encoded by CDKN2a and p14ARF encoded by an alternative reading frame of CDKN2a [21]. There is often co-deletion of these loci in most of cancers in human. The present study is first comprehensive study of these proteins and ADFP in human renal cancer tissues. Experimental data suggests CDKN2AB −/− knockout mice develop aggressive and multiple tumours - suggesting tumour suppresser role of these proteins. Our observation and previously published study [22] confirms the expression of p16 protein is absent or low in renal cell cancer samples, suggesting that loss of the p16 gene may be key event involved in renal cell cancer. Cyclin-dependent kinase (CDK) inhibitor p16^INK4a^ protein is a member of the INK4 family of cell cycle regulatory proteins and specifically inhibits the formation of cyclin D1-CDK4/6 complexes [23, 24]. The later controls the activity of retinoblastoma tumor-suppressor protein pRb by phosphorylation. Phosphorylated pRb then disassociates from the E2F transcription factor family of proteins. The E2F transcription factors are then able to reprogram the cell to enter the S phase [25]. Dephosphorylated retinoblastoma protein arrests proliferating cells in G1 phase of the cell cylce. From the published literature, it is reasonable to assume that the cellular response to hypoxia, a common feature in renal cancers involves reversible cell cycle arrest characterized by dephosphorylated Rb, loss of CDK activity, and decreased cyclin synthesis. We also study expression of ADFP, a protein encoded by 9p21 and reported by us previously. The expression of protein correlated with 9p deletion and grade of cancer. Also, its expression correlated with two other known proteins such as CAIX and p16. In contrast to previous studies [26, 27], CAIX was not found to be associated with aggressive tumours or long-term prognosis.

## Conclusions

In conclusions, the study has confirmed previous findings that p21 region harbours one of the tumour suppressor genes and the loss of heterozygosity in this region is associated with aggressive tumours and predicted poor clinical outcomes on long-term. The loss of expression of cell-cycle related proteins and ADFP is associated with aggressive cancers.

## Abbreviations

ADFP, adipose differentiation protein; CAIX, carbonic anhydrase 9; CDK, cyclin-dependent kinases; DNA, deoxyribose nucleic acid; DSS, disease specific survival; HR, hazard ratio; I-FISH, interphase fluorescent in situ hybridization; RCC, renal cell carcinoma; RFS, recurrence free survival

## Additional file

Additional file 1: Figure S1. Microsatellites analysis showing allelic deletion in tumour 9, 81 and 103 involving more than one marker (note differences in the red box). (JPG 85 kb)
