# Supplementary figures and images for: Microsatellite alteration and immunohistochemical expression profile of chromosome 9p21 in patients with sporadic renal cell carcinoma following surgical resection
Source: BMC Cancer. 2016 Jul 27;16:546. doi: 10.1186/s12885-016-2514-8 (PMC4963937; doi:10.1186/s12885-016-2514-8)

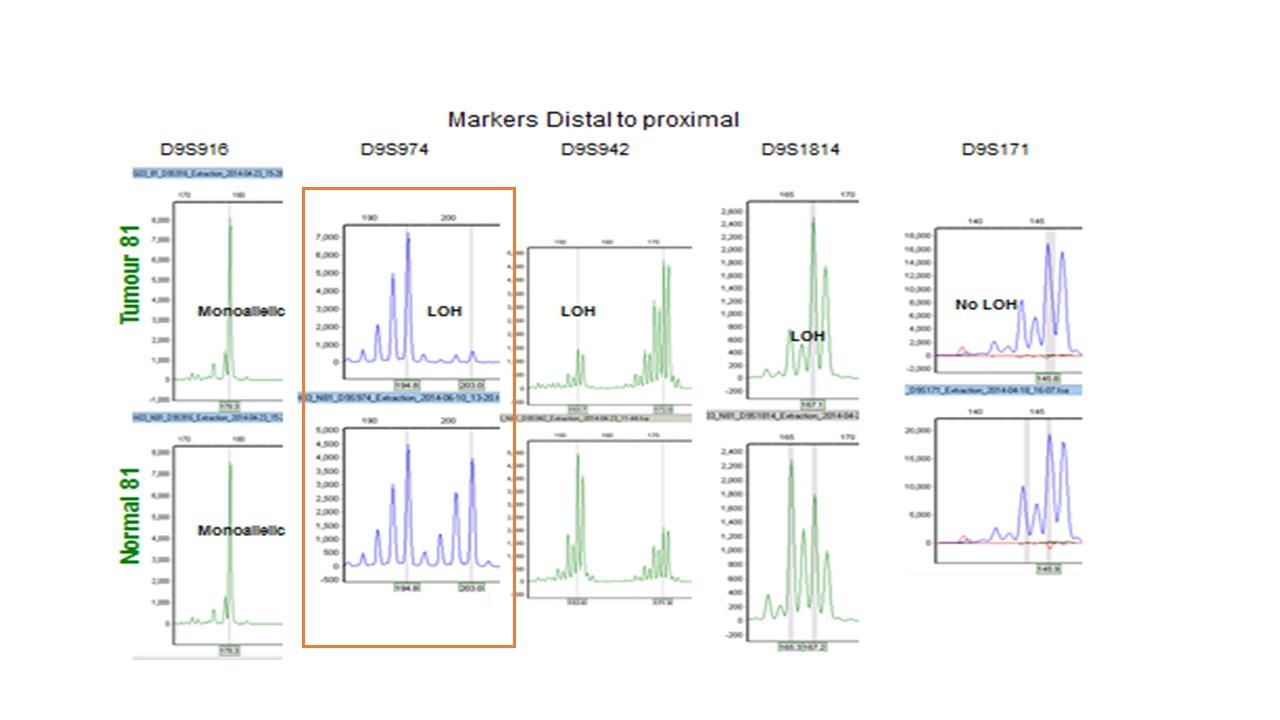

Supplement: Additional file 1: Figure S1. — Microsatellites analysis showing allelic deletion in tumour 9, 81 and 103 involving more than one marker (note differences in the red box). (JPG 85 kb) [file 12885_2016_2514_MOESM1_ESM.jpg]
